# Predictors of thromboembolic complications after stent-assisted coiling of acutely ruptured intracranial aneurysms: A retrospective multicenter study

**DOI:** 10.3389/fcvm.2022.922858

**Published:** 2022-08-03

**Authors:** Gaozhi Li, Haixia Xing, Guohua Mao, Jing Cai, Dianshi Jin, Yujie Tian, Xiaohua Zhang, Bing Zhao

**Affiliations:** ^1^Department of Neurosurgery, Renji Hospital, Shanghai Jiao Tong University School of Medicine, Shanghai, China; ^2^Department of Pathology, Shanghai East Hospital, Tongji University School of Medicine, Shanghai, China; ^3^Department of Neurosurgery, The Second Affiliated Hospital of Nanchang University, Nanchang, China; ^4^Department of Neurosurgery, Linyi People's Hospital, Linyi, China; ^5^Department of Neurosurgery, Dalian Central Hospital, Dalian, China

**Keywords:** intracranial aneurysm, ruptured aneurysm, endovascular treatment, stent-assisted coiling, thromboembolic complication

## Abstract

**Background:**

Stent-assisted coiling (SAC) has been reported to safely and effectively treat wide-necked unruptured intracranial aneurysms. However, SAC of acutely ruptured aneurysms is controversial because of perioperative thromboembolic complications. We aimed to investigate the predictors of the thromboembolic complications after SAC of acutely ruptured aneurysms.

**Methods:**

We performed a retrospective multicenter analysis of 110 consecutive patients with ruptured intracranial aneurysms treated with SAC within 72 h of the onset of subarachnoid hemorrhage. Thromboembolic complications were defined as any angiographic filling defects at the aneurysms base or the distal artery during the stent treatment and the new onset of symptomatic ischemia and a new hypo-density in a vascular distribution confirmed by CT scan within 24 h of treatment. These patients were grouped into patients with thromboembolic complications and those without thromboembolic complications. A multivariate logistic regression analysis was performed to identify predictors of thromboembolic complications.

**Results:**

One hundred and one patients with 101 ruptured aneurysms were included in this study. 9 (8.9%) patients experienced thromboembolic complications. Patients with thromboembolic complications had a higher rate of unfavorable outcomes at discharge (*P* < 0.001) and at the last follow-up (*p* = 0.017). Of these patients, four patients presented with intraprocedural thrombus formation, and 5 experienced postprocedural ischemia. There was a trend toward thromboembolic complications in patients with a higher Fisher grade (*p* = 0.076) and those treated with intravenous tirofiban (*p* = 0.052). Patients with thromboembolic complications more often presented with poor grade clinical conditions (*p* = 0.005) and aneurysms with a large dome to neck ratio (*p* = 0.031). In the multivariate analysis, a worse World Federation World Federation of Neurological Societies (WFNS) grade (OR = 8.241; 95% CI 1.686–40.292; *P* = 0.009) and a larger dome to neck ratio (OR = 5.385; 95% CI 1.023–28.337; *P* = 0.047) were independent predictors of thromboembolic complications.

**Conclusion:**

Patients with thromboembolic complications are more likely to have an unfavorable outcome. A worse clinical condition before the treatment and a larger dome to neck ratio were independent predictors of thromboembolic complications after SAC of acutely ruptured intracranial aneurysms.

## Introduction

Aneurysmal subarachnoid hemorrhage (aSAH) is a fatal hemorrhagic stroke with a 30-day mortality rate of 45%, and about 30% of survivors have moderate to severe disability (1). Surgical clipping and endovascular treatment are essential treatment modalities to prevent rebleeding after a ruptured aneurysm. Endovascular treatment has a lower incidence of unfavorable outcomes than surgical clipping and has become an alternative treatment for ruptured aneurysms (2).

With the development of neurointerventional technologies, stent-assisted coiling (SAC) has been used to treat wide-necked unruptured intracranial aneurysms with a relatively low recurrence rate at follow-up angiography. SAC of ruptured aneurysms may be feasible, safe, and effective in current studies (3, 4). However, periprocedural complications more often occur in SAC of ruptured aneurysms than unruptured aneurysms or single coiling of ruptured aneurysms (3–6). Stents usually require dual antiplatelet therapy concerning hemorrhage, especially with bleeding from the external ventricular drainage or placement of a ventriculoperitoneal shunt (2, 7). Moreover, the thrombotic events are the most common complications after SAC of ruptured aneurysms and are associated with increased mortality (6, 8–10). Therefore, the SAC of acutely ruptured aneurysms is still controversial (2, 7). Identifying predictors of thromboembolic complications is vital for patients' safe treatment.

The Low-profile Visualized Intraluminal Support (LVIS) device is self-expanding nickel titanium, single-wire braid, retrievable, closed-cell microstent (8). The pivotal US LVIS trial results have shown that the LVIS stents allow safe and highly effective coiling of wide-necked aneurysms (11). However, the LVIS stents with higher metal coverage seem to cause more common thromboembolic complications than others, including Neuroform and Enterprise stents (9, 12). In this study, we performed a retrospective multicenter analysis of SAC of ruptured aneurysms within 72 h of the onset of subarachnoid hemorrhage with LVIS stents. We aimed to investigate the predictors of thromboembolic complications after SAC of acutely ruptured aneurysms.

## Methods

### Study design and patients

The institutional review board approved this study, and the informed consent was waived. This retrospective multicenter study focused on patients with acutely ruptured aneurysms treated with LVIS stents (13). One hundred and ten consecutive patients with ruptured aneurysms performed SAC within 72 h of the onset between January 2017 to December 2017. Four centers were tertiary hospitals with more than 150 intracranial aneurysms each year. We collected clinical characteristics, including demographic information, medical history, personal history, World Federation of Neurosurgical Societies (WFNS) grade before the treatment, Fisher grade, clinical treatment reports, aneurysm characteristics, periprocedural complications, and clinical outcomes.

### Endovascular treatment protocol

Endovascular treatment was considered in patients with ruptured aneurysms whose characteristics were suitable for coiling or clipping, or those with posterior circulation aneurysms. SAC was used for wide-necked aneurysms (neck size ≥4 mm or dome to neck ratio ≤2). All ruptured aneurysms were treated by LVIS (Microvention/Terumo, Tustin, CA). These procedures were performed under general anesthesia. A 50–75 IU/kg bolus of heparin was used for heparinization. The stent was manipulated by semi-jailing technology of partially deploying to cover the aneurysm neck. External ventricular drainage (EVD) was often considered in patients with acute hydrocephalus or severe intraventricular hemorrhage (IVH) after the treatment. Patients were transferred to the neurosurgical intensive care unit (NICU) and treated with standard management for vasospasm.

### Antiplatelet management

There was no standard protocol for antiplatelet therapy, and the regimens were prescribed according to institutional standards in four medical centers. Two different protocols were used in this study. One is a loading dose of 300 mg clopidogrel and 300 mg aspirin administered through a nasogastric tube or rectally 2 h before the stent deployment. The other is a loading dose of tirofiban (8–10 ug/kg) infused intravenously during the stent deployment. Tirofiban was maintained at 0.10 ug/kg/min for at least 12 h after the procedure. If thromboembolic complications occurred, 5–10 ml of tirofiban were infused through the microcatheter, and immediate angiography was checked every 5–10 min. Dual antiplatelet therapy (100 mg of aspirin and 75 mg of clopidogrel) was prescribed daily. The platelet function testing was not routinely measured.

### Thromboembolic complications definition

The primary outcome was the thromboembolic complication. The thromboembolic complications were defined as angiographic filling defects at the aneurysms base or the distal artery during the stent treatment and the new onset of symptomatic ischemia and a new hypo-density in parent and distal arteries distribution in the first 24 h after the procedure (13). Angiograms and CT scans were reviewed by both independent interventional neuroradiologist and neurosurgeon who were not involved in the treatment. These patients were grouped into patients with thromboembolic complications and those without thromboembolic complications.

### Clinical outcome measurement

Clinical outcomes at discharge and the last follow-up were measured using a modified Rankin Scale (mRS). The favorable outcome was defined as a mRS of 0–2. The mean time of follow-up was 15.9 ± 10.4 months.

### Statistical analysis

Statistical analysis was performed with SPSS 22.0 (IBM SPSS; Armonk, NY, US). Continuous data were presented as mean ± standard deviations, and categorical variables were given as frequency (percentage). The Chi-square test and Fisher's precision test were used for categorical variables. The *t*-test and Wilcoxon's rank-sum test were used for continuous variables. We compared demographic information, clinical characteristics, and outcomes between patients with thromboembolic complications and those without thromboembolic complications group. Univariate and multivariate logistic regression analyses were performed to identify the predictors of thromboembolic complications. Variables with a *p*-value < 0.1 in univariate analysis were entered into the multivariate analysis using the backward method. Odds ratio (OR) and 95% confidence interval (CI) were calculated. A *p*-value < 0.05 was considered to be statistically significant.

## Results

### Baseline characteristics

Out of the 110 patients, 101 patients with 101 ruptured aneurysms were included in this study. Three patients with carotid artery blood blister aneurysms, 4 with dissection aneurysms, and 2 with multiple aneurysms were excluded. The mean time of SAC of aneurysms was 31.6 ± 18.8 h of subarachnoid hemorrhage. 49 (48.5%) patients were treated within 24 h. 9 (8.9%) patients experienced thromboembolic complications. Of these patients, four patients experienced intraprocedural thrombus formation, and 5 experienced ischemia after treatment. Baseline characteristics between patients with thromboembolic complications and those without thromboembolic complications groups are presented in Table 1. There was no statistically significant difference in age, gender, medical history, angiographic vasospasm, aneurysm location, and the neck size of ruptured aneurysm between the two groups.

**Table 1:** Baseline characteristics between patients with thromboembolic complications and those without complications.

**Variables**	**Thromboembolic** **complications (*****n*** = **9)**	**Without thromboembolic** **complications (*****n*** = **92)**	***P*** **-value**
Age (years, ± SD)	61.8 (10.4)	58.0 (10.4)	0.296
No. (male/female)	9 (1/8)	92 (32/60)	0.180
Current smoking (%)	0 (0)	21 (22.8)	0.998
Hypertension (%)	6 (66.7)	48 (52.2)	0.411
WFNS grade (%)			0.005
Grade I-III	5 (55.6)	84 (91.3)	
Grade IV-V	4 (44.4)	8 (8.7)	
Fisher grade (%)			0.076
Grade I-II	4 (44.4)	68 (73.9)	
Grade III-IV	5 (55.6)	24 (26.1)	
Angiographic vasospasm (%)	2 (22.2)	20 (21.7)	0.973
Multiple aneurysms (%)	2 (22.2)	14 (15.2)	0.586
**Aneurysm location (%)**			
ACA-ACoA	1 (11.1)	24 (26.1)	0.340
PCoA-ICA	6 (66.7)	56 (60.9)	0.734
MCA	2 (22.2)	6 (6.5)	0.120
Posterior circulation	0 (0)	6 (6.5)	0.999
Aneurysm size (mm, ± SD)	5.8 (2.6)	4.5 (2.7)	0.185
Neck size (mm, ± SD)	3.8 (1.4)	3.7 (1.6)	0.814
Dome to neck ratio	1.6 (0.5)	1.2 (0.4)	0.031
**Antiplatelet regimens (%)**			
Aspirin and clopidogrel	5 (55.6)	77 (83.7)	0.052
Tirofiban	4 (44.4)	15 (16.3)	0.052

WFNS, World Federation of Neurological Society; ACA, anterior cerebral artery; ACoA, anterior communicating artery; PCoA, posterior communicating artery; ICA, internal carotid artery; MCA, middle cerebral artery.

### Clinical outcomes

Clinical outcomes at discharge and follow-up are presented in Table 2. The last follow-up was available for 100 (99%) patients. 84 (83.2%) patients achieved a favorable outcome (mRS 0–2) at discharge. Patients with thromboembolic complications more often had a higher rate of unfavorable outcomes at discharge (*P* < 0.001) and at the last follow-up (*p* = 0.017). There was no statistically significant difference in the mortality rate between the two groups (*p* = 0.999).

**Table 2:** Clinical outcomes between patients with thromboembolic complications and those without complications.

**Clinical** **outcomes**	**Thromboembolic** **complications** **(*****n*** = **9)**	**Without** **thromboembolic** **complications (*****n*** = **92)**	***P*** **-value**
**mRS at discharge**			
mRS0-2	3 (33.3%)	81 (88%)	0.001
mRS3-5	6 (66.7%)	9 (9.8%)	<0.001
mRS6	0 (0)	2 (2.2%)	0.999
**mRS at follow-up**			
mRS0-2	6 (66.7%)	83 (91.2%)	0.039
mRS3-5	3 (33.3%)	6 (6.6%)	0.017
mRS6	0 (0)	2 (2.2%)	0.999

mRS, modified Rankin Scale.

### Predictors of thromboembolic complications

Results of the univariate and multivariate analyses of predictors of thromboembolic complications are presented in Table 3. There was a trend toward thromboembolic complications in patients with a higher Fisher grade (*p* = 0.076) and treated with intravenous tirofiban (*p* = 0.052). Patients with thromboembolic complications presented with a WFNS grade of IV-V (*p* = 0.005) and a larger dome to neck ratio (*p* = 0.031). In the multivariate analysis, poor WFNS grade (OR = 8.241; 95% CI 1.686–40.292; *p* = 0.009) and a larger dome to neck ratio (OR = 5.385; 95% CI 1.023–28.337; *p* = 0.047) were independently associated with the presence of thromboembolic complications.

**Table 3:** Univariable and multivariable analyses of thromboembolic complications.

**Variables**	**Univariable**		**Multivariable**	
	**OR (95% CI)**	***p*** **-value**	**OR (95% CI)**	***p*** **-value**
WFNS grade	8.4 (1.871–37.704)	0.005	8.241 (1.686–40.292)	0.009
Fisher grade	3.542 (0.878–14.286)	0.076		
Dome to neck ratio	5.607 (1.174–26.781)	0.031	5.385 (1.023–28.337)	0.047
Antiplatelet regimen	4.107 (0.986–17.099)	0.052		

WFNS, World Federation of Neurological Society; CI, confidence interval.

## Discussion

We performed a retrospective multicenter analysis of patients with acutely ruptured intracranial aneurysms treated with stents. We found that patients with thromboembolic complications more often had an unfavorable outcome. There was a trend toward thromboembolic complications in patients with a higher Fisher grade and those treated with intravenous tirofiban. A worse clinical condition and a larger dome to neck ratio were independently associated with the presence of thromboembolic complications. These findings suggest that selected patients with a good clinical grade and a small dome to neck ratio could be safely treated with SAC with a lower risk of thromboembolic complications.

We found that thromboembolic complications occurred in 8.9% of patients treated with SAC within 72 h of hemorrhage. This complication is not rare in the treatment of acutely ruptured aneurysms. Our finding is similar to previous studies that showed the incidence of thromboembolic complications was 7.8–15% after SAC of ruptured intracranial aneurysms (14–17). Bsat et al. (18) systematically reviewed the literature on SAC of acutely ruptured aneurysms in 1,582 patients and found that the overall rate of thromboembolic complications was 9.1% (95% CI: 6.0–12.7%; I = 72.8%). There was a high rate of thromboembolic complications, probably because of insufficient coverage of antiplatelet therapy or antiplatelet resistance (19). Recently, Xue et al. (20) reported that ischemic procedure-related complications occurred in 3 (7.5%) of 40 patients with 40 ruptured middle cerebral artery aneurysms with glycoprotein IIb/IIIa inhibitor for the LVIS stents treatment of ruptured aneurysms. Meanwhile, previous studies have shown that thromboembolic complications increase procedural disability (6, 8, 9). We also found unfavorable clinical outcomes more often in patients with thromboembolic complications.

We found that a poor-grade clinical condition (WFNS grade of IV or V) was an independent predictor of thromboembolic complications. Imamura et al. (6) also reported that a poor grade condition on admission was a risk factor for ischemic complication during endovascular embolization of ruptured aneurysms. An elevated D-dimer concentration was observed in most patients with ruptured aneurysms, especially in patients with a poor-grade SAH. Elevated D-dimer levels on admission independently had a higher risk of thromboembolic events (21).

We found that ruptured aneurysms with a large dome to neck ratio were associated with increased thromboembolic complications. Liu et al. (22) also reported periprocedural thromboembolic complications were more likely to occur in ruptured aneurysms with a smaller dome to neck ratio. However, there was no statistically significant difference. Our previous study regarding predicting intraprocedural complications during coiling of ruptured anterior communication artery aneurysms showed that aneurysm neck size and parent vessel angle were independent predictors of thrombus formation (10). The definitive relationship between aneurysm morphology and periprocedural complication is still unknown.

We found no significant difference in thromboembolic complications in both antiplatelet therapy regimens. There was a trend toward thromboembolic complications in patients with intravenous tirofiban. Kim et al. (23) conducted a retrospective analysis of patients who underwent SAC of ruptured aneurysms using intravenous tirofiban instead of loading doses of dual antiplatelet agents. They found that tirofiban may be an effective and safe alternative with a low risk of thromboembolic complications. However, there is no standardized antiplatelet management for SAC of ruptured aneurysms, and the DELPHI consensus statement prefers a dual-antiplatelet regimen with aspirin and a glycoprotein IIb/IIIa inhibitor (24).

There is a paucity of data on antiplatelet management for SAC of acutely ruptured aneurysms, and the relationship between antiplatelet therapy regimens and thrombotic complications requires further study. P2Y12 reaction test could predict thrombotic complications, but its value is uncertain. The P2Y12 test to guide clopidogrel therapy did not decrease the incidence of thromboembolism complications (25). Kim et al. (26) found that high antiplatelet drug resistance was associated with postprocedural infarction after coiling for unruptured aneurysms. Li et al. (27) conducted a retrospective analysis of patients who received modified antiplatelet therapy using thromboelastography (TEG) after SAC of ruptured aneurysms, and they found no significant difference in the rates of thromboembolic events between individualized antiplatelet therapy with TEG parameters and standard dual antiplatelet therapy without TEG test groups. Their tests were performed on the 3rd day after treatment of ruptured aneurysms (27). In our study, nearly half of the patients were treated within 24 h, and the time-costing test is challenging for treating acutely ruptured aneurysms.

Our study has several limitations. First, this was a retrospective multicenter study. Antiplatelet regimens vary widely. Thromboelastogram or drug sensitivity gene tests for platelet were not routinely examined in the emergency department in these centers. Second, we included symptomatic ischemia. The incidence of asymptomatic ischemia may be underestimated because diffusion-weighted imaging was not routinely performed within 24 h. Third, aneurysm morphology may affect the incidence of thromboembolic complications. We did not yet identify the exact procedure reasons, including vasospasm after stimulation during the operation of the catheter and guide wire or thrombosis in the catheter due to insufficient irrigation water during the procedure in this retrospective study. Besides, all aneurysms were treated with LVIS stents. LVIS seems to cause more common procedural-related thrombotic events than the other stents (9, 12). There is variability in choice and techniques of stent type. Whether this finding is generalized to other stents still requires further study. Nevertheless, these data represent the stent treatment of acutely ruptured aneurysms and may be more helpful in guiding the treatment decision.

## Conclusion

We conducted a multicenter cohort of patients with acutely ruptured intracranial aneurysms treated with SAC. Patients with thromboembolic complications more often had an unfavorable outcome and presented with a poor WFNS grade. Selected patients with a good clinical grade and a small aneurysm dome to neck ratio could be safely treated with SAC with a lower thromboembolic complication rate.

## Data availability statement

The original contributions presented in the study are included in the article/supplementary material, further inquiries can be directed to the corresponding author/s.

## Ethics statement

The studies involving human participants were reviewed and approved by the Institutional Review Board of Renji Hospital, Shanghai Jiao Tong University School of Medicine. Written informed consent for participation was not required for this study in accordance with the national legislation and the institutional requirements.

## Author contributions

GL and HX wrote the first manuscript. GM, JC, DJ, YT, and BZ were involved in the acquisition and analysis of the data. HX and BZ contributed to the statistical analysis. XZ and BZ were involved in the conceptualization of the study. All authors were involved in data interpretation and approved the final manuscript.

## Funding

This work was supported by Shanghai Science and Technology Project (21Y11906200), National Facility for Translational Medicine (Shanghai) TMSK-2021-147.

## Conflict of interest

The authors declare that the research was conducted in the absence of any commercial or financial relationships that could be construed as a potential conflict of interest.

## Publisher's note

All claims expressed in this article are solely those of the authors and do not necessarily represent those of their affiliated organizations, or those of the publisher, the editors and the reviewers. Any product that may be evaluated in this article, or claim that may be made by its manufacturer, is not guaranteed or endorsed by the publisher.
